# Mining Public Opinions on COVID-19 Vaccination: A Temporal Analysis to Support Combating Misinformation

**DOI:** 10.3390/tropicalmed7100256

**Published:** 2022-09-22

**Authors:** Victor Diogho Heuer de Carvalho, Thyago Celso Cavalcante Nepomuceno, Thiago Poleto, Jean Gomes Turet, Ana Paula Cabral Seixas Costa

**Affiliations:** 1 Eixo das Tecnologias, Campus do Sertão, Federal University of Alagoas, Delmiro Gouveia 57480-000, Brazil; 2 Núcleo de Tecnologia, Centro Acadêmico do Agreste, Federal University of Pernambuco, Caruaru 55014-900, Brazil; 3 Departamento de Administração, Federal University of Pará, Belém 66075-110, Brazil; 4 Departamento de Engenharia de Produção, Federal University of Pernambuco, Recife 50740-550, Brazil

**Keywords:** COVID-19, pandemics, vaccination, Brazil, opinion mining, temporal analysis, twitter data, misinformation

## Abstract

This article presents a study that applied opinion analysis about COVID-19 immunization in Brazil. An initial set of 143,615 tweets was collected containing 49,477 pro- and 44,643 anti-vaccination and 49,495 neutral posts. Supervised classifiers (multinomial naïve Bayes, logistic regression, linear support vector machines, random forests, adaptative boosting, and multilayer perceptron) were tested, and multinomial naïve Bayes, which had the best trade-off between overfitting and correctness, was selected to classify a second set containing 221,884 unclassified tweets. A timeline with the classified tweets was constructed, helping to identify dates with peaks in each polarity and search for events that may have caused the peaks, providing methodological assistance in combating sources of misinformation linked to the spread of anti-vaccination opinion.

## 1. Introduction

Digital social networks have become popular channels for news dissemination and individual opinion sharing given the ease of access and speed of circulation of free information, being one of the most striking reflexes in people’s daily use of Internet resources [1]. The social web can be seen as a crossroads of institutional communication strategies, in other words, as a place of discussion capable of containing daily debate and anticipating these social debates, encouraging multiple points of view [2] of hundreds of millions of users posting content daily [3]. However, this same ease of access has repercussions in the distortion of information and the dissemination of misinformation [4,5].

The coronavirus disease 2019 (COVID-19) outbreak brought the world the need for non-pharmaceutical sanitary measures and mobility interventions such as social isolation with physical distancing, which made work from home a reality while waiting for vaccines to ensure a safe return to normal activities [6,7]. Managers (whether in the public or private spheres) needed to learn to manage uncertainties to keep organizational activities running while dealing with different risks [8], especially at the personnel level.

The increasing use of online communication platforms generated a large volume of information about the pandemic and related online social behavior, including behaviors that were harmful to the general welfare due to the virality with which information is propagated through the social web [9]. Twitter, for example, was one of the social networks where the spread of messages about the spread of COVID-19 around the world was most noticeable [10].

The presented facts have significant repercussions for the organizational use of this information, as reported in the related literature, and we can summarily assume that:

- Organizations have a rich information environment for decision-making, especially regarding people’s opinions present in online discussions on the social web;
- As a negative aspect of this rich environment, organizations have to deal with ungenuine information, in other words, with the dissemination of misinformation capable of affecting social welfare and causing impacts on people’s lives.

This scenario brought a wave of false information about COVID-19 vaccines in Brazil, where several prominent people from the national media, including artists, politicians, and popular social network influencers, affected public opinion about the vaccination [11]. This movement was noticed through social networks, generating bans and blockades for some of these personalities for their continuous presentation of information of dubious origin or fake news related to COVID-19 [12,13,14].

The dissemination of false information through the social web, in general, became the target of official criminal investigations and inquiries, promoted either by Brazilian Legislative and Judiciary powers to find their sources and apply the necessary measures to contain the problem [15,16]. The need for mechanisms to detect and refute rumors that can cause social harm has become even more evident in this pandemic context, full of public figures propagating information that is often unverified [17,18].

From the perspective of sociopolitical analysis, policymakers want to know about public responses to specific topics and events related to some political issue, which makes it essential to assess which events may be associated with significant movements in public opinion [19].

This article intends to present the results of a study based on a temporal analysis of public opinions about COVID-19 immunization in Brazil using tweets, natural language processing, and machine learning for this purpose.

The following goals can be derived from this objective:

1. To present the general composition of the corpora and general timeline according to the tweets collected.
2. To test machine learning classification models and select the one with the best performance for multiclass classification tasks.
3. To present the distribution of opinions on vaccination against COVID-19 on a timeline, identifying neutral, pro-, and anti-vaccination peaks.
4. Based on a search in social web news channels, identify possible events causing the movements in opinions according to peak dates.

## 2. Methods

An opinion analysis framework can describe the process applied in this work based on other analytical processes proposed in the literature (see, for instance, [20,21,22,23]). Essentially, a framework or analytical process for sentiment or opinion analysis has to deal with [24,25]:

(a) Data collection to assemble the corpora.
(b) Text cleaning and preprocessing.
(c) Training and testing the sentiment classification models (for instance, machine learning).
(d) Best model selection.
(e) Polarity annotation for each text in a corpus.

**Figure 1:**
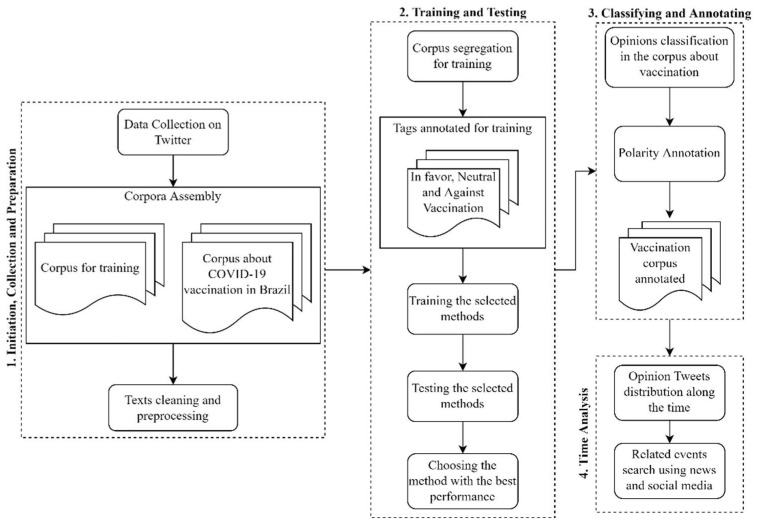
Workflow for public opinion analysis about the COVID-19 vaccination in Brazil.

The framework implemented and applied in our research contains all these steps, but it adds one more item: the temporal opinion analysis. Figure 1 presents the workflow in our framework, and the subsequent subsections describe the four-part process.

### 2.1. Initiation, Data Collection, and Preparation

Twitter API 2.0 for academic research ensured a more comprehensive collection of data for constructing both corpora, limiting the search and retrieval to tweets of Brazilian origin. A scraper was developed using Python’s “requests” library to connect the API and make the necessary requests to retrieve the tweets.

A strategy following those adopted by Oliveira et al. [26] and de Carvalho et al. [27] was applied to assemble the training corpus: using sets of hashtags (#) in the Portuguese language containing expressions related to pro (in favor) and anti (against) vaccination contents and neutral contents (news or informative contents) observed on Twitter users’ conversations. Table 1 presents the segregation of these hashtags (in Portuguese) according to their alignment. Note that the sets are not exhaustive.

The tweets were stored in comma-separated values (csv) format to be accessed as a corpus containing fields such as the tweet id, the text, and post creation data. The csv files were aggregated in a unique file using the Pandas library [28] for data analysis.

The corpus containing tweets about vaccination in Brazil, for classification, was collected based on a specific period of seventeen months—from June 2020 to October 2021—covering the six months after the beginning of the vaccination campaign in Brazil and the ten months of the vaccination campaign. The csv file that contains this corpus has the same tweet information fields as the training corpus file. This second corpus was obtained using several keywords and hashtags related to COVID-19 and the vaccine, including the previous ones presented in Table 1.

Table 2 contains the terms (in the Portuguese language) used in the queries, separated by kind, except the terms already presented in Table 1 and used to search for tweets in this second corpus.

For all queries constructed with the terms in Table 2, two statements were added, one regarding the country (*place_country:BR*) defining Brazil as the source of tweets. The other was to set Portuguese as the language of the tweets to be collected (*lang:pt*). Sets of terms with the designation “free term” were defined to search for tweets with any of these terms in the textual composition, considering that they could have hashtags. In the case of the sets of terms with “hashtags”, the search sought only the defined ones.

Text cleaning and preprocessing consisted of case folding; eliminating punctuation, emojis/emoticons, numbers, stop words, and links/URLs; tokenization; and lemmatization. Cleaning and preprocessing were performed using a script developed based on the SpaCy library [29] in Python [30], which contains a series of functions implemented.

The texts in both corpora were vectorized in two forms: using term frequency-inverse document frequency (TF-IDF) vectorizer and count vectorizer applying pre-built functions from Scikit-Learn library [31]. The vectors were built considering n-grams among uni-grams, bi-grams, and tri-grams. Additionally, in both corpora, duplicity removal was not applied since duplicity in this study was considered a support to an initial opinion posted by a user and endorsed by others, for instance, as a “retweet” repassing the original message to reach more significant users.

### 2.2. Training and Testing Models

Training and testing processes were applied at the document level with the preprocessed and vectorized texts considering six supervised machine learning classification methods: multinomial naïve Bayes, logistic regression, linear support vector machines, random forests, adaptative boosting, and multilayer perceptron. All methods were imported from the Scikit-Learn library.

The naïve Bayes classifier is an algorithm based on Bayes theorem, calculating a posterior probability, assuming independence among the classified attributes [32]. The multinomial naïve Bayes (MNB) classifier applied in this study works with the frequency of words, considering feature vectors represented by entire indications of a repeated word, not just the presence of the word, which corrects weaknesses in the original method [33].

Equation (1) describes how this algorithm’s posterior probability is calculated [34]:

(1) $$P\left(c\;|\;d\right)=P\left(c\right)\overset{\left|d\right|}{\underset{i=1}{\prod }}P{\left(w_{i}|c\right)}^{f_{i}^{d}}$$

In Equation (1), *P*(*c*) in the prior probability of class *c*, and $P{\left(w_{i}|c\right)}^{f_{i}^{d}}$ is the probability of a word *w_i_* belonging to a class *c*, this probability being elevated to the number of occurrences of the word in document *d*, calculated by $f_{i}^{d}$.

The logistic regression (LogReg) classifier is a linear regression algorithm to make predictions when the dependent variable is binary [35]. The algorithm uses a function to minimize the estimators’ errors using the log-likelihood and applies gradient descent to determine the parameters that produce the best estimators [36]. For the classification task, according to Mitroi et al. [37], given a set of documents *d_i_*, where *y^i^* is the class of a document, de logistic regression maps the documents to classes using a sigmoid function *h_θ_* (*d_i_*) to determine the parameters of the vector $\theta =\left\{\theta_{0},\theta_{1}\ldots ,\theta_{m}\right\}\;$ fitting the regression line describer by Equation (2):

(2) $${\hat{y}}^{i}=h_{\theta }\left(d_{i}\right)=\frac{1}{1+e^{-\theta^{T}\left[1-d_{i}\right]}}$$

The linear support vector machines classifier (LinearSVC), according to Cichosz [38], belongs to the most effective general-purpose machine learning classification algorithms. It also applies binary classification, and the class predictions are determined according to a hyperplane as in Equation (3):

(3) $$h\left(x\right)=w\cdot x+b$$

In Equation (3), *w* is a weights vector, *x* is the input vector for training, and *b* is the bias. Equation (4) represents the optimization problem in the linear support vector machines classifier [39]:

(4) $$min_{w,b,\varepsilon }\;\frac{1}{2}w^{T}w+C\;\overset{I}{\underset{i=1}{\sum }}\varepsilon^{i}$$

In Equation (4), *w^T^* is the weights vector, and *i* represents the class, with restrictions: $y_{i}\left(w\cdot x+b\right)-1\;\geq 0,\;\forall \;i=1,\;\ldots ,\;n$.

These three first algorithms are considered baseline classifiers for comparing with the most sophisticated/complex classifiers, such as the next three presented.

The random forests (RF) classifier is an ensemble strategy bootstrapping algorithm based on the decision trees algorithm [40]. This algorithm constructs multiple decision trees in a random subspace of feature space. In each subspace, the unpruned tree generates the final step classifications and combines all the decisions generated for the final prediction [41,42]. The intricate formulation of the random forests algorithm can be seen in Breiman [43].

The adaptative boosting classifier (ADA) is another ensemble algorithm that uses a boosting technique for constructing a strong classifier, combining weak classifiers [44], and according to Sharef et al. [45], the function with the linear combination of these classifiers is given by Equation (5):

(5) $$f\left(x\right)=\overset{T}{\underset{t=1}{\sum }}\alpha_{t}h_{t}\left(x\right)$$

In Equation (5), *α_t_ h_t_* (*x*) is a weighted weak classifier that will take training where each *x* belongs to the same domain *X*, having a related label *y* from a set *Y*.

The multilayer perceptron (MLP) is a neural network classification algorithm that learns a function $f\left(\cdot \right)=R^{m}\;\to \;R^{o}$ using a training dataset in which *m* and *o* are the numbers of dimensions for the input and the output, respectively [46]. The MLP procedure is divided into forward and backward propagations using the backpropagation algorithm and is used to generalize a nonlinear function demonstrated in Equation (6) [47]:

(6) $$f\left(X\right)=b_{2}+W_{2}\times \left(f_{A}\;\left(b_{1}+W_{1}+X\right)\right)$$

In Equation (6), the two *W* are the weight matrices of the hidden layer and the output layer; the two *b* represent bias vectors of the hidden and output layers; the *f_A_* is the activation function.

All machine learning methods were trained and tested using the vectorized texts, with each n-gram defined with each type of vectorization method. Fivefold cross-validation was applied to identify each method’s performance metrics regarding their classification accuracies. The classification reports, the construction of each method’s ROCs, and their confusion matrices were obtained using Scikit-Learn’s model selection and metrics functions. The metrics obtained through the classification report were precision, recall, f1-score, and accuracy. The formulas for each metric are given from Equations (7) to (10):

(7) $$Precision=\frac{TP}{TP+FP}$$

(8) $$Recall=\frac{TP}{TP+FN}$$

(9) $$F1=2\times \frac{Precision\times Recall}{Precision+Recall}$$

(10) $$Accuracy=\frac{TP+TN}{TP+TN+FP+FN}$$

In Equations (7), (8), and (10): *TP* is the number of True-positive classifications; *FP* is the number of False-positive classifications; *TN* is the number of True-negative classifications; *FN* is the number of False-negative classifications.

### 2.3. Classification and Annotation

It was possible to apply the classification of tweets in the corpus using the best method according to the evaluations made in the training and tests stage, later making their annotation according to the tags assigned by the classifier. This process generated an update in the corpus file to introduce the column referring to these tags, leaving the corpus ready for the subsequent analyses.

### 2.4. Time Analysis

The temporal analysis focused on presenting variations in the number of tweets according to the multiclass classification, seeking to identify specific dates with notable class variations. A manual search was carried out through the social web to find news about events possibly influencing the detected variations.

## 3. Results

### 3.1. Corpora Composition and Time Series

Initially, 143,615 tweets were collected for the first corpus, but some performance issues occurred when training models with this number of texts, namely a lack of computational memory to complete training. Therefore, random cuts were applied to both classification cases to leave the corpus with a number of tweets that, during classification, would still guarantee a memory slack for further computations. These cuts resulted in a new total of 49,494 tweets, now distributed according to Table 3.

The second corpus, explicitly created to receive the final opinion classification process, contains 221,884 tweets. The primary information in this corpus is the tweet identification number, text, and creation date.

**Figure 2:**
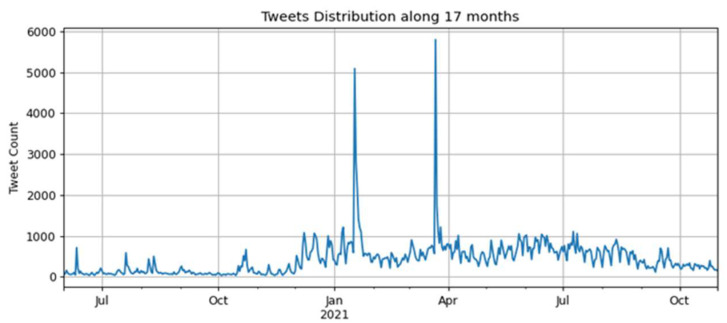
Time series of tweets over seventeen months, from June 2020 to October 2021.

The time series containing the daily amounts of tweets for the second corpus is presented in Figure 2, considering seventeen months, from 1 June 2020 to 31 October 2021.

The collection of tweets considered seventeen months:

- One semester before the start of vaccination against COVID-19—the period from June to December 2020.
- The remaining eleven months run from January 2021, when the first vaccine against COVID-19 was applied in Brazil, until October.

In Figure 2, there was an increase in tweets between December 2020 and January 2021 after the Brazilian federal government launched the national COVID-19 vaccination campaign in December 2020. There were two peaks in the number of tweets, occurring between January and February 2021 and between March and April 2021, surpassing 5000 tweets.

In the interval between January and February 2021, the first peak can be explained by the beginning of the application of the first vaccine shot on 17 January 2021, in the state of São Paulo. In the interval between March and April 2021, when the second peak occurred, there was a presidential announcement in a national broadcast on 23 March 2021 about the COVID-19 pandemic and the measures related to the vaccination campaign in Brazil that may have caused this high number of tweets.

The number of tweets retrieved about the vaccination in the first semester of 2021 was higher than in the second semester of 2020, demonstrating that the vaccination campaign may have influenced people to comment more on the social web, specifically on Twitter. The following section will present the performance results of the models used based on training and testing using the collected tweets.

### 3.2. Training and Testing Results

Training and test rounds separated the first corpus into two parts: the training part with 75% of the set and the testing part with 25%. Two kinds of vectorization were applied to the tweets’ texts: the typical count of frequencies and the term frequency–inverse document frequency (TF–IDF), generating two great results sets for six different models, each with three different n-grams.

#### Models’ Performances

The classification models are directly related to the objective of this study, and selecting the best one is a fundamental step in the analytical process. The scores obtained in the training–testing process are shown in Table S1 in the Supplementary Materials, considering both vectorizers and all n-grams.

**Figure 3:**
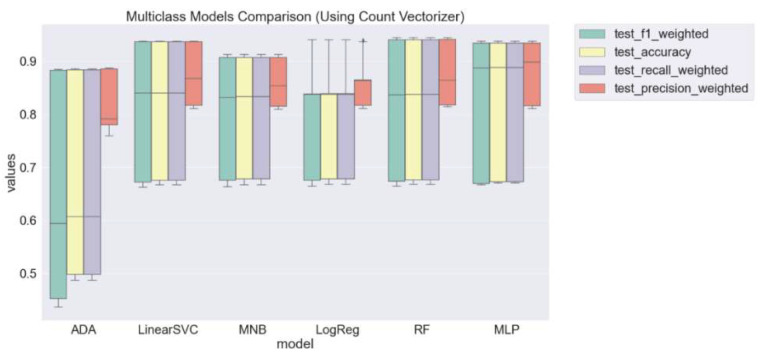
Overall weighted scores for each model trained and tested with count vectorizer.

The RF with TF–IDF vectorizer and uni-grams presented the best accuracy (0.95), followed by RF (with count vectorizer and uni-grams), LogReg, LinearSVC, and MLP, both with the two vectorizers and uni-grams, with accuracies of 0.94. Figure 3 contains the boxplots for the overall performances of models with count vectorizer considering the weighted scores.

**Figure 4:**
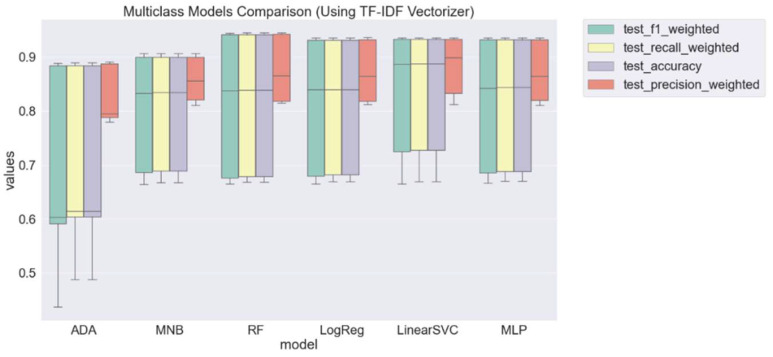
Overall weighted scores for each model trained and tested with TF–IDF vectorizer.

The aggregated results for the model using count vectorizer demonstrate that LinearSVC, RF, and MLP have very close performances with a soft advantage of RF. Figure 4 contains the results using the TF–IDF vectorizer.

**Figure 5:**
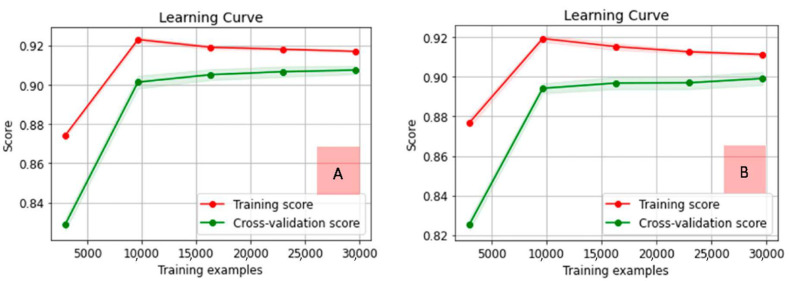
Learning curves for MNB with count (**A**) and TF–IDF (**B**) vectorizers and uni-grams.

Looking for a model with the best trends in learning curves, MNB for both vectorizers and uni-grams presented a behavior with better control regarding overfitting. Figure 5 illustrates the learning curves for MNB (both vectorizers).

**Figure 6:**
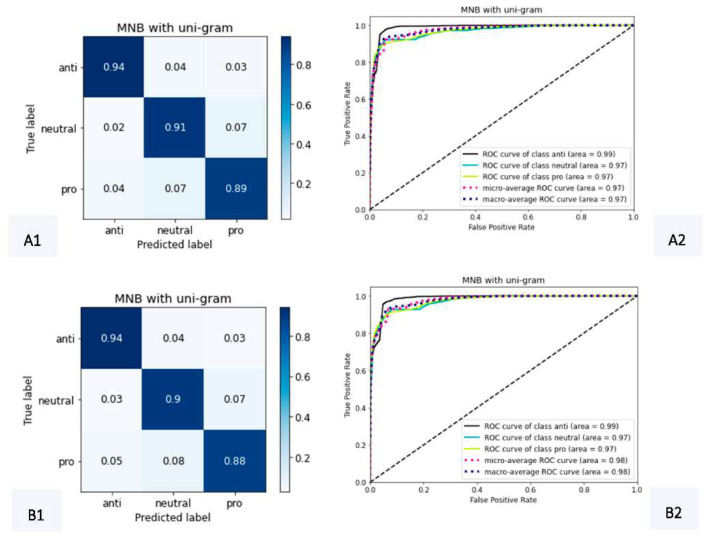
Confusion matrices and ROC curves for MNB with count (**A1**,**A2**) and TF–IDF (**B1**,**B2**) vectorizers, using uni-grams.

Learning curves for both MNB cases are similar. The count vectorizer model is slightly superior and tends to converge faster than the same model using TF–IDF, between 0.90 and 0.92, close to 30,000 examples. For both vectorizers, accuracy started below 0.88 for training and below 0.84 for cross-validation curves, but with close to 10,000 examples, training curves achieved their peaks, softly decreasing until close to 30,000; moreover, cross-validation kept increasing tendencies after 10,000 until 30,000 examples. Confusion matrices and ROC curves for MNB cases in the analysis are presented in Figure 6.

Confusion matrices indicated that MNB with count vectorizer had slightly higher true positive percentages, and both ROC curves demonstrated good-quality models, with AUCs about 97% of correct classifications. Although MNB models for both cases presented the best behavior in learning curves, both cases had AUCs slightly smaller than RF (with 99%). These conditions of better control on overfitting led to the preference of MNB over RF since we wanted to reduce the noise caused by overfitting on the results as much as possible.

More information on selecting the best model can be found in the Supplementary Materials, through Figures S1–S4 and related comments.

### 3.3. Distribution of the Classified Tweets over the Time

The classification applied over the second corpus using MNB generated a distribution of tweets according to the opinion polarity shown in Table 4. The number of tweets classified as neutral was the highest, even surpassing the sum of the pro- and anti-vaccination tweets: There is a percentage of 53.47% of neutral tweets, while the other two classes have 46.53% together.

**Figure 7:**
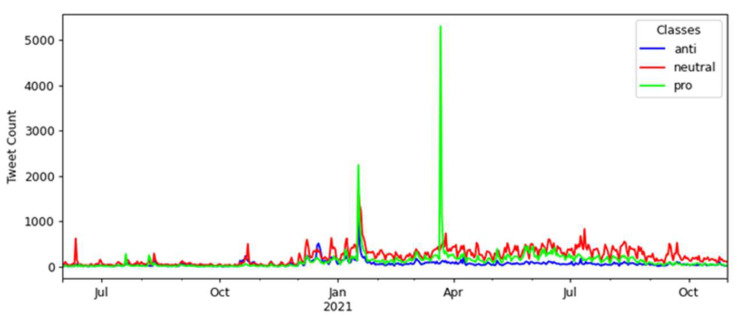
Tweets’ polarity distribution over 17 months about the COVID-19 vaccination.

The opinion polarity timeline from June 2020 to October 2021 is presented in Figure 7, classifying tweets into “neutral”, “pro-”, and “anti-vaccination” according to the best model selected in the previous section.

There were two notable spikes in tweet numbers over the 17 months: the first between January and February 2021 and the second between March and April 2021. In these two intervals, news and social media searches gave us evidence of events that may have been associated with these peaks (in italic de descriptions of the identified events, referring to 2021 dates):

- 1st peak in a month interval which contains four events:
  - *Brazilian President’s online broadcast on COVID-19 awareness* on 7 January.
  - *The first COVID-19 vaccine was applied in Brazil* on 17 January.
  - *A speech by the Brazilian President about CoronaVac* on 22 January.
  - *The Brazilian President confirmed that the government had approved the purchase of a COVID-19 vaccine by private companies* on 25 January.
- 2nd peak in a month interval which contains three events:
  - *The law’s enactment authorized the federal government to join the Covax Facility* on 2 March.
  - *The Brazilian President confirmed Pfizer’s vaccine purchase with the news that the first shots will arrive in April* on 4 March.
  - *A presidential radio transmission announced that Brazil would be self-sufficient in producing COVID-19 vaccines* on 23 March.

In both intervals, the number of pro-vaccination tweets was higher than in the neutral and anti-vaccination classes, demonstrating the periods where there was greater support for vaccination in Brazil via Twitter throughout the timeline considered in this study. The first interval presents a much larger distribution of tweets in the pro-vaccination class, with 2244, followed by the neutral class, with 1690 tweets, and having its smallest amount in the anti-vaccination class, with 1157 tweets. These numbers refer to 17 January 2021, corresponding to *the first COVID-19 vaccine applied in Brazil*. In this case, public opinion registered on Twitter tended to support COVID-19 vaccination.

**Figure 8:**
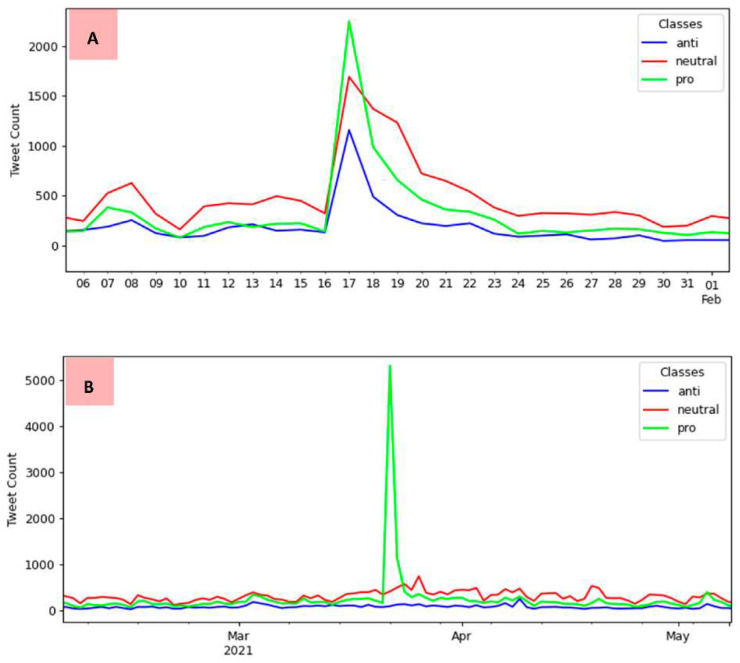
Details of the peaks: (**A**) refers to the first interval peak and (**B**) to the second interval peak.

Also notable is the trend of support for vaccination in the second interval, with a peak of 5301 pro-vaccination tweets. For neutral tweets, the number was 409, while for anti-vaccination tweets, it was even lower, reaching just 88 tweets. This condition occurred on 22 March 2021, the closest date recorded in the events to the *presidential radio transmission, which announced that Brazil would be self-sufficient in producing COVID-19 vaccines* on 23 March. Figure 8 provides a closer view of each period when these peaks occurred.

In looking for evidence of some remarkable event that took place on 22 March 2021, a tweet was found by the Brazilian President informing that “Brazil would be the fifth country that most applied vaccines”. This fact was refuted by the press when it was found that the data presented referred to “the total number of vaccines per 100 inhabitants, being counted as a single dose and may not be equal to the total number of people vaccinated, depending on the specific dose regimen, for example, in the case of people receiving multiple doses” [48].

Expressly for 17 January 2021, it can be noted that the theme of the tweets revolves around the beginning of the COVID-19 immunization campaign in Brazil. Table 5 contains three examples of tweets’ texts (one for each class) posted on that day. Elements other than text have been removed, such as references to other users (with @), emoticons, and URLs.

The first text in Table 5 communicates the application of the first vaccine in Brazil. In contrast, the second text registers an acknowledgment concerning the application of the vaccine, having a positive intonation about the necessary care against the disease and celebrating the related science and public institutes. The third exemplified text demonstrates a negative opinion about one of the vaccines being applied in Brazil.

Investigating even more about possible events that may have influenced the spike in posts on 22 March 2021, there was a milestone in the COVID-19 pandemic in Brazil since the country surpassed the total of 12 million confirmed cases on this date [49]. Another event that can be considered concerns the release by the Brazilian federal government that states and municipalities could use the vaccines already delivered to date to apply the first shot to people who had not yet received it [50]. The last example that may be related was the news of the bill that mandated presenting proof of COVID-19 vaccination to attend events in person in public or private establishments liable to agglomeration [51].

A valuable resource for helping to detect which events caused the peak in the number of tweets on 22 March was topic modeling and extraction, as in Cotfas et al. [21]. However, the related process was not applied in this research, remaining as a recommendation for future work. Table 6 also presents three examples of tweet texts, one from each class, these referring to 22 March.

In Table 6, the texts follow the same trend as in the previous table: The first text presents data on vaccination; the second text contains an acknowledgment about scheduling a person to receive the vaccine; and the third text contains an opinion following a denial trend—the use of early treatment with the “COVID kit”—even though the person claims that he will be vaccinated.

**Figure 9:**
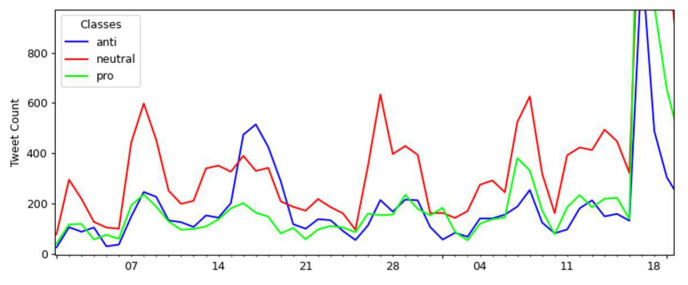
Details of the interval between December 2020 and January 2021.

An interesting interval of months for a brief analysis is between December 2020 and January 2021. The Brazilian federal government launched the COVID-19 national vaccination campaign during this period, where an increase in the number of tweets on the topic can be noted compared with previous months. More details about this specific period can be seen in Figure 9.

The period recorded in the details of Figure 9 starts on 1 December 2020 and ends on 18 January 2021. On 8 December is recorded a small spike of 600 neutral tweets, with 246 anti-vaccination tweets and 236 pro-vaccination tweets.

In this interval, the following events occurred (in italic a description of the events, referring to 2020 dates):

- *The official televised launch of the COVID-19 National Vaccination Campaign* on 16 December.
- *A speech by the Brazilian President about the application of vaccines to the Brazilian population* on 18 December.
- *An online broadcast of the Brazilian President where he talked about COVID-19 and vaccination* on 24 December.
- *A speech by the Brazilian President about laboratories needing to register vaccines to sell to Brazil* on 28 December.

The event that occurred on 16 December may be related to the spike in anti-vaccination tweets between 14–17 December. This event was the official release of Brazil’s COVID-19 National Vaccination Campaign. On 16 December, the number of anti-vaccination tweets was 474, increasing to 515 on the next day and falling to 425 on 18 December. This day had the second-most anti-vaccination tweets registered in the months of the collection, just behind 17 January 2021.

On 28 December, there was a small peak of 634 neutral tweets that coincided with a speech by the Brazilian President on the issue of registering vaccines for sale in Brazil. Another little peak in neutral tweets occurred on 8 January 2021, with 626 registers. A day before (7 January), the Brazilian President had talked about awareness against COVID-19 in an online broadcast. On 7 January, also there was an increase in the number of neutral tweets.

**Figure 10:**
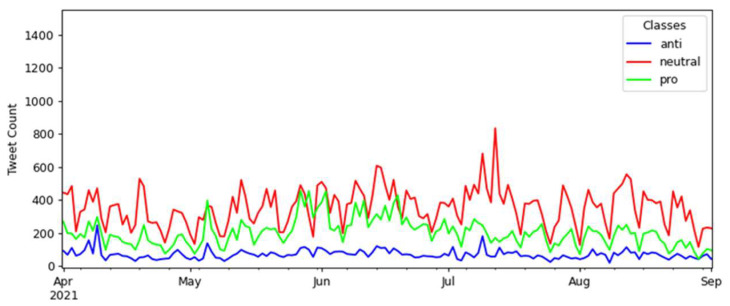
Details of the interval between the beginning of April and the end of August 2021.

The interval between the beginning of April and the end of August 2021 also presents interesting behaviors concerning tweet counts about vaccination. Figure 10 contains a cutout of this period.

Notably, there was a peak of 834 neutral tweets on 12 July, plus a higher trend of posts maintaining neutrality. It is also notable that there is always a trend toward more pro-vaccination tweets regarding pro and anti-vaccination tweets. The 12 July peak comprises the following events (descriptions in italic):

- *A deponent who allegedly lied received a prison order by the COVID’s Parliament Inquiry Commission* on 7 July.
- *The Brazilian President left the hospital after days of hospitalization, talking about the use of drugs against the COVID-19* on 18 July.

With the further investigation of events associated with the pandemic that took place on 12 July, it is observed that the Brazilian press reported that Brazil had registered the lowest number of cases of COVID-19 since January 2021 [52] and that vaccination in Brazil had reached more than 115 million vaccines applied, according to a survey by the national press consortium [53].

The highest pro-vaccination tweet counts in this period occurred successively on 27 May, with 456 tweets; on 29 May, with 454 tweets; and on 2 June, with 453 tweets. The following events occurred in the same period (description in italic):

- *The Brazilian President says the country will have a monthly record for distributing vaccines against COVID-19* on 22 May.
- *The Brazilian President emphasizes vaccination and criticizes isolation in an official statement* on 2 June.

The two events described in the previous paragraph incorporate the three dates with the highest number of pro-vaccination tweets in their range of occurrence. Notably, the second event occurred on the date of the third-highest pro tweet count (2 June).

It is necessary to emphasize that the period between December 2020 and October 2021 has other occurrences that may be interesting for a more detailed analysis of events associated with the pandemic, with more significant variations in the numbers of tweets between the classes of opinions studied. The distribution of tweets over the 17 months over which the collection was carried out demonstrates a general trend of more neutral posts, followed by pro-vaccination, with anti-vaccination posts generally having minor numbers in both daily and total counts.

#### Summary of the Events Search on News and Social Web According to the Opinion’s Polarity Timeline

**Figure 11:**
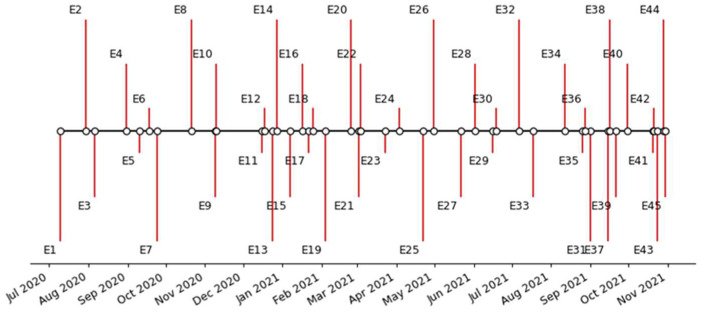
Events timeline.

We identified several possibly related events with the tweets’ collection. Table S2 in the Supplementary Materials contains a list of 45 events and dates (provided as month/day/year), some previously mentioned. Figure 11 contains the timeline of the 45 events (coded from E1 to E45).

As can be noted in Table S2, what makes Brazil different from other anti-vaccination movements worldwide is the federal government’s recurrent position in several moments supporting the sharing and spreading of anti-vaccination information [54].

## 4. Discussion

In general terms, the reported study aimed to demonstrate the power of support that tools from machine learning and data analysis, especially using texts, can provide so that authorities can combat misinformation through the social web. Several works developed in other countries corroborate this power, for instance:

- Batra et al. [20] analyzed the sentiments expressed in tweets concerning COVID-19 vaccination in six countries: India, Pakistan, Norway, Sweden, Canada, and United States;
- Cotfas et al. [21] used a corpus of tweets in English to analyze public opinion about the COVID-19 vaccine in the United Kingdom;
- Luo et al. [55] used posts from Twitter and Sina Weibo to analyze public perceptions of COVID-19 vaccination in the United States and China;
- Alliheibi et al. [56] analyzed Saudi citizens’ opinions about vaccines.

The training and testing process demonstrated that the MNB using the count vectorizer and uni-grams had the best trade-off related to the overfitting issue and the correctness of the applied classifications. Regarding the classifications’ correctness, in addition to the metrics presented in Table S1 in the Supplementary Materials, the ROC curves were presented, and the respective AUCs were considered, as can be seen in Figure 6, explicitly referring to MNB. Regarding overfitting, the learning curves presented in Figure 5 helped to understand the occurrence of this phenomenon, also supporting the selection of the method with the best performance.

These findings do not mean that MNB is the best model for all situations: The model testing process we demonstrated is necessary for choosing the best fit according to characteristics related to precision and correctness using the dataset available, avoiding or at least reducing as best as possible problems involving generalization, such as overfittings [57].

We were able to demonstrate a process for identifying potential events related to peaks in the number of messages containing polarized opinions about the vaccination campaign against COVID-19 in Brazil, following premises we observed in previous studies [20,21,22,58] and opening space for continuities by applying other techniques capable of further filtering useful information by collecting messages from social network users. The analytical process we applied demonstrates that it is possible to generate mechanisms for automating searches for news of benign or harmful events to verify if they were the focus of misinformation or fake news [5,59].

The constructed timelines supported manual searches of social web news channels to find records of events that may have caused, on specific dates, peaks in any of the classes of opinions. Temporal analysis is an instrument to support epidemiologic analysis, considering the context of “infodemic”, literally a large amount of misinformation harming something by its power of influence over people [60], such as what occurred in the COVID-19 vaccination campaign in Brazil. An interesting complement to this kind of analysis is the use of geographic information, performing spatio-temporal analysis such as that described by da Silva et al. [61] and Cunha et al. [62], developed in Brazil in epidemiological and vaccinal contexts, respectively.

From a practical point of view, this work serves as a reference for regulatory organizations, or even public health and public security management agencies, for tracking sources or foci of misinformation causing hesitancy in people still unvaccinated. It should be noted that the power of identifying news spread across the social web increases with machine learning and text mining tools. In a greater scope, the analysis presented in our study can support other research, such as that by Guo et al. [63], towards creating incentive policies to increase the vaccination rate.

From the perspective of text mining, given that texts potentially referring to fake news were found, named entities such as people, dates, and places [64,65] can be identified, ensuring the traceability of the sources. This process can be used to identify, for instance, people who originated or spread misinformation or fake news [66], ensuring that authorities adopt measures to contain their actions.

This subject proves to be relevant at a global level, as demonstrated by Lin et al. [60]. They investigated how government-sponsored misinformation campaigns have significantly affected the expansion of epidemic situations worldwide, using data from 149 countries from 2001 to 2019. They identified that misinformation was significantly associated with the incidence and prevalence of water-respiratory diseases in more susceptible populations in the analyzed period.

Considering the general context of the COVID-19 pandemic, several tools from artificial intelligence, mainly machine learning ones, have been used to support the fight against misinformation, as demonstrated by Galhardi et al. [15], citing some examples found: Jain and Kasbe [67] proposed a method for detecting fake news using posts from Facebook users applying the naïve Bayes classifier; Faustini and Covões [68] studied fake news detection in three different languages (Portuguese, English, and Bulgarian), testing several fake news corpora with four machine learning methods (naïve Bayes, k-nearest neighbors, support vector machines and random forests); and Rangel et al. [66] presented an author profile task using long short-term memory with posts from Twitter in two languages (English and Spanish) to identify fake news authors.

Another task related to artificial intelligence, sentiment analysis, is used to access people’s sentiments and opinions about several aspects of the COVID-19 pandemic. To give a few examples: Bhat et al. [69] demonstrated the applicability of sentiment analysis using posts from Twitter to understand the social media response to the COVID-19 outbreak; Shorten et al. [57] surveyed deep learning applications to support combating the current pandemic, and found applications in public sentiment analysis about the critical world health situation; Melton et al. [70] developed a public sentiment analysis about the COVID-19 vaccines using textual data extracted from Reddit.

In this last case, to emphasize the relevance of understanding public sentiments and opinions about the COVID-19 vaccination, Alamoodi et al. [71] developed a survey about the theme. They found 33 articles and identified data sources, specific kinds of sentiment or opinion mining applications, and methods used for the analysis, highlighting, among others, machine learning, deep learning and transfer learning methods, natural language processing frameworks, and topic modeling.

The development of systems for identifying sources of misinformation by the information technology departments associated with these public authorities may consider tools such as those used and tested in the study reported here, promoting robust mechanisms, either in real-time or very close to it, for capturing misinformation. Rigorously trained corpora, going through the entire process of eliminating noises that affect identifications, are essential elements, being the starting point for the actions of the bodies responsible for monitoring and containing harmful events related to the COVID-19 vaccination campaign and any other disease for which there is a vaccine.

Brazil, for example, already has information systems to deal with situations such as vaccination campaigns, registering vaccinated people, and allowing a view over time and geographic space of the population’s vaccination cycles [62]. This information held by health authorities could be cross-referenced with information extracted from social networks about what people have expressed and how they feel about vaccination so that necessary steps can be taken to ensure the construction of positive public opinion, correcting any harmful effect that the disinformation propagated by the same social networks may have caused.

Finally, it is also important to note that the applicability of the analytical components in this study is not limited to the horizon of vaccination campaigns. There are several possibilities for analyzing public opinions and sentiments on health management and disease combatting.

### Technical Difficulties and Challenges

Specifically related to vaccination, there are initiatives in favor of corpus constructions to enable analysis (see, for instance, [9,70]). This tendency within the pandemic context is one of the most prominent due to the adverse effects that misinformation can produce [72]. Some techniques and methods are needed for texts’ collection, initial treatment, and sentiment or opinion classification and for analyzing the data in the corpora. This process involves textual data scraping and natural language processing techniques so the texts can be preprocessed and cleaned, eliminating noises that could hinder the application of classification techniques, such as machine learning [73,74].

The technical process we developed and implemented for the data collection on Twitter was not free of difficulties, problems, and challenges. We had to deal with several constraining elements, mainly considering the limitations of the Twitter API. As commented in Section 2.1, the collection process considered the sets of terms shown in Table 1 (for the first corpus) and Table 2 (for the second corpus).

Specifically for the second corpus, we intended to efficiently retrieve as many results (tweets) as possible, considering a constricted scraping with the search terms defined, delimiting the process only to retrieve tweets from Brazil and Portuguese during the period presented in the timeline. In this case, an efficient way means avoiding a time-consuming process as best as we can.

We also had other concerns about developing our scraping process since there are constraints on the number of results to be returned. Consulting the Twitter Developers online community (this community can be accessed through the URL: https://twittercommunity.com/, accessed on 17 September 2022) and Stack Overflow forums (the Stack Overflow forums can be found through the URL: https://stackoverflow.com/, accessed on 17 September 2022), we defined two critical parameters in our main scraping script: the number of tweets to be retrieved per request (*max_results*) and the number of request rounds (*max_request_rounds*).

The number of results per request was defined as the maximum allowed (*max_results = 500*). The number of request rounds was defined as 3000 (*max_request_rounds = 3000*), and these parameters hypothetically would allow us to retrieve a total of 1,500,000 tweets. Despite this possible maximum number, we could retrieve 221,884 in the implemented process, so we admitted that this number was the total considering all the restrictions of terms, geographic location, language, and time we imposed.

It is also vital to comment on another concern during this process: we had 10,000,000 tweets allowed by Twitter API 2.0 for academic projects. When we performed the collection, this amount was divided for testing/retesting processes and the final collection, not only for the research we reported in this article. The division was thought to guarantee precisely the collection of the most extensive possible amounts of text for each parallel research in development, considering that the maximum number would only be restored at the beginning of the next monthly cycle.

This whole challenging context has significant repercussions for the sampling of our study under two main considerations:

1. From the training process perspective, related to the first corpus with 143,615 tweets, we assumed the hypothesis that this number of texts ensured the reliability of the selected classification methods since, for this case, the sample was based on the number of textual components (or features) among uni-grams, bi-grams, and tri-grams.
2. Especially for the hashtags’ sets presented in Table 1, for the first corpus construction, despite not being exhaustive, they were defined to ensure the retrieval of representative numbers of tweets for each pro, anti, and neutral part of the corpus, providing a training corpus also with significant numbers of considered features. Table 3 separates the initial numbers according to the polarities and the final number after a random cut for balancing the number of texts for each polarity class.
3. For the second corpus, we assumed the hypothesis that the 221,884 tweets represented the maximum number existing for the 17 months to which the retrieval was applied, considering the constraints we imposed on the process. Note that we had parametrized the script to retrieve 1,500,000 results.

## 5. Final Considerations

This article reports a study involving machine learning models and text mining to analyze public opinion expressed by tweets over 17 months on COVID-19 vaccination in Brazil. The study showed, therefore, the entire process so that the analysis could be carried out, building at the end a timeline with the variations in the numbers of “neutral”, “pro”, and “anti” vaccination tweets, in addition to trying to carry out a match between “pro” and “anti” tweets as subjective opinions, and “neutral” as objective texts, referring to the transfer of information and news communication. With the entire framework built, the central idea is to ensure that the public power agencies responsible for monitoring actions related to the vaccination campaign can identify events that cause misinformation.

We achieved interesting levels of precision in the tested methods using the built corpora using the MNB method with unigrams and the count vectorizer. This method presented promising results for the metrics used, as well as presenting excellent precision for the areas under the curve (AUCs) referring to the three classes: 99% for the class “anti”, 97% for “pro” and for “neutral”.

### 5.1. Limitations

Even with several essential preprocessing tasks, there may still be enough text noise to cause distortions in tests performed with machine learning methods and, consequently, in the classifications. Minimizing this noise is a process that must be considered critical when preprocessing texts to ensure that subsequent classifications are accurate.

The number of tweets should also be increased to ensure a more significant representation and power of describing the phenomenon being studied (misinformation). One of our biggest concerns and problems was reaching an adequate number of texts to analyze. For more practical applications, developing a tweet scraping strategy that guarantees a more significant number of texts, their quality, and alignment with the explored theme is imperative. An enhanced scraping strategy should also consider our comments on the difficulties and challenges in the retrieval process.

Another critical aspect that was not considered in our study was the separation of genuine tweets from fake ones posted by bots. This would have involved new layers in the analytical process, such as authorship identification and the detection of bots between real (human) authors to exclude their posts.

We also realized that a hierarchical classification process is necessary to ensure proper alignment with the idea that opinions classified as pro- and anti-vaccination are subjective (in other words, they represent opinions) and not objective (which could represent some communication of some occurrence or some news).

The event identification process we applied in our research was also quite limited. Although we were able to create a timeline since the first discussions about vaccination in Brazil, verifying the variations in the numbers of tweets according to each polarity (pro, anti, and neutral), the process of identifying the events potentially influencing the number of tweets about the vaccination opinions posted was manual. A search was carried out in news channels about the dates observed in the mentioned timeline, where the value curves underwent some notable changes for a more dedicated investigation. It is possible to automate these searches using machine learning tools combined with the extraction of entities (names of people and organizations and even dates).

Monselise et al. [23] give an example of direction for the event identification process using topic modeling. Their study was directed at the public sentiment about COVID-19 immunization in the United States using Twitter posts. They applied VADER and BERT for sentiment and emotion classification (“positive”, “neutral”, and “negative”), and online non-negative matrix factorization (ONMF) for topic modeling. They could identify trending topics reflecting public concern about the vaccination, and respective responses to the topics through the polarity of emotions and sentiments, stating that misinformation is one of the major causes of negative emotions.

The psychological impact of COVID-19 vaccination is another significant issue to be investigated, as indicated by Babicki et al. [75]. These authors analyzed the impact of the vaccination on the security sense, anxiety, and quality of life of Polish people with a sample of 1677 respondents to their data collection instrument; about 25.4% were vaccinated at least the first dose, and 17.9% declared that they not only did not take the vaccine but also had no intention of getting vaccinated. They found that vaccination reduces anxiety levels about being infected and the presence of infected people in the same environment. People who do not intend to be vaccinated adopt a denialist attitude towards the pandemic, including anti-vaccination behavior, with a significantly low level of anxiety about COVID-19.

### 5.2. Further Research

We can indicate directions for further research based on all that we have presented regarding the implications and limitations: (i) formulate a refined strategy for scraping the social web, for example, Twitter, to guarantee a more significant number of texts, which also ensures its thematic alignment and quality; (ii) adopt a hierarchical classification strategy aiming at a better alignment between the pro- and anti-vaccination classes with the idea that they are subjective, that is, they actually represent people’s opinions and not news or the objective communication of information about some event; (iii) improve the strategy for detecting events capable of generating misinformation, considering the period under study and named entities; (iv) apply topic modeling and extraction, also to help identify keywords for use in searches referring to the previous item; (v) use geoinformation and metrics data from tweets to generate a spatio-temporal analysis of public opinions on COVID-19 vaccination in Brazil; (vi) improve the data–event correlation identification strategy; (vii) identify authorship followed by bot detection between authors, to exclude fake posts from bots; and (viii) measure COVID-19 vaccination psychological impact on the Brazilian population.

## Figures and Tables

**Table 1:** Positional hashtags about the COVID-19 vaccination in Brazil.

Pro	Neutral	Anti
*#vacinaja, #vacinaparatodos, #vacinabrasil, #vacinaurgenteparatodos, #vacinasim, #vemvacina, #vacinaprageral, #vacinaemgeral, #queremosvacina*	*#vacina, #vacinacao, #vacinacorona, #vacinacovid, #vacinacoronavirus, #vacinacovid19, #vacinacovid_19, #vacinacaocovid*	*#vacinanao, #eunaovoutomarvacina, #eunaosoucobaia, #naovousercobaia, #vacinaobrigatorianao, #naovoumevacinar*

The italicized terms are in Portuguese.

**Table 2:** Terms used to retrieve tweets for the corpus for classification.

**COVID and pandemic-related hashtags**	*#covid, #covid-19, #covid19, #covid_19, #coronavírus, #pandemia*
**COVID and pandemic-related free terms**	*covid, covid-19, covid19, covid_19, coronavirus, pandemia*
**Vaccination-related hashtags**	*#vacina, #vacinacao, #vacinacovid-19, #vacina_corona, #vacina_covid, #vacina_covid-19, #vacina_covid_19, #vacina_coronavirus, #vacina_covid19, #vacinacaocorona, #vacinacaocovid-19, #vacinacaocovid_19, #vacinacaocoronavirus, #vacinacao_corona, #vacinacaocovid19, #vacinacao_covid, #vacinacao_coronavirus, #vacinacao_covid19, #vacinacao_covid-19, #vacinacao_covid_19*
**Vaccination-related free terms**	*vacina, vacinacao, vacinar, vacinado*

The italicized terms are in Portuguese.

**Table 3:** Training and testing corpus tweet counts before and after random cuts.

Before Random Cut		After Random Cut	
Tags	Count	Tags	Count
Pro	49,477	Pro	16,498
Anti	44,643	Anti	16,498
Neutral	49,495	Neutral	16,498
Total	143,615	Total	49,494

**Table 4:** Amounts and percentages of tweets classified according to the tags.

Tag	Count	%
Anti	34,700	15.64
Neutral	118,645	53.47
Pro	68,539	30.89
Total	221,884	100.00

**Table 5:** Examples of tweets’ texts from 17 January 2021.

Tweets’ Texts	Translation	Class
*Que fique registrado que a primeira vacina de covid-19 no Brasil foi aplicada a contragosto do governo federal*	Let it be noted that the first COVID-19 vaccine in Brazil was applied contrary to the federal government’s support	Neutral
*Graças a Deus… Que está vacina seja abençoada na vida de cada ser humano!!! E continuemos com os cuidados… Viva a ciência e viva os institutos públicos.*	Thanks to God… May this vaccine be blessed in the life of every human being!!! And let’s continue with the care… Long live science and long live public institutes.	Pro
*Respeito o seu ponto de vista! Acho que é cedo para se tirar alguma conclusão sobre qualquer vacina. Mais essa Coronavac a procedência não me traz confiança afinal na China que essa pandemia começou!*	I respect your point of view! I think it is too early to draw any conclusions about any vaccine. But the precedence of this Coronavac does not give me confidence, after all, it was in China that this pandemic started!	Anti

Italicized sentences are in Portuguese.

**Table 6:** Examples of tweets’ texts from 22 March 2021.

Tweets’ Texts	Translation	Class
*A Secretaria de Saúde vacinou 1.502 idosos com a 1ª dose da vacina contra a covid-19 nesta segunda, 22. O município também aplicou a segunda dose do imunizante em 46 trabalhadores de saúde e 10 idosos. Desde o início da campanha, 30.795 pessoas foram vacinadas com a 1ª dose*	The Health Department vaccinated 1502 older adults with the 1st shot of the COVID-19 vaccine on Monday, 22. The municipality also applied the second shot of the immunizing agent to 46 health workers and 10 older adults. Since the beginning of the campaign, 30,795 people have been vaccinated with the 1st shot	Neutral
*Gratidão a Deus! Vacina agendada para paaaai. O coração transborda de emoção só pelo agendamento, imaginem quando ele se vacinar!!!*	Gratitude to God! The vaccine is scheduled for my father. The heart overflows with emotion just by scheduling, imagine when he gets vaccinated!!!	Pro
*Não ser obrigatória a vacina é respeitar os direitos individuais! Qdo chegar minha vez eu vou tomar a vacina, e tenho meu kit covid comprado.*	Vaccination is not mandatory, it means respecting individual rights! When my turn comes, I will get the vaccine and have my covid kit purchased.	Anti

Italicized sentences are in Portuguese.

## Data Availability

The corpora files are not publicly available because of Twitter’s Developer Agreement; however, we can make them available upon request and for academic research purposes.

## Supplementary Materials

The following supporting information can be downloaded at: https://www.mdpi.com/article/10.3390/tropicalmed7100256/s1, Table S1: Models’ performances for the classification test, Table S2: Some notable events throughout the COVID-19 pandemic in Brazil and their dates, Figure S1: Learning curves for RF with count vectorizer and uni-grams, Figure S2: Confusion matrix and ROC curves for RF with count vectorizer and uni-grams, Figure S3: Learning curves for RF with TF–IDF Vectorizer and uni-grams, Figure S4: Confusion matrix and ROC curves for RF with TF-IDF Vectorizer and uni-grams.
