# Supplementary material for: Mining Public Opinions on COVID-19 Vaccination: A Temporal Analysis to Support Combating Misinformation
Source: Trop Med Infect Dis. 2022 Sep 22;7(10):256. doi: 10.3390/tropicalmed7100256 (PMC9607799; doi:10.3390/tropicalmed7100256)
Supplement: Supplementary file 1 [file tropicalmed-07-00256-s001.zip › tropicalmed-1868047-supplementary.pdf]

Supplementary Materials

Supplementary Tables

Table S1. Models' performances for the classification test.

Model	n-gram	Tag	Count Vectorizer				TF-IDF Vectorizer			
			Prec.	Rec.	F1	Acc.	Prec.	Rec.	F1	Acc.
		pro	0.92	0.93	0.93		0.91	0.94	0.92	
	uni-gram	anti	0.90	0.90	0.90	0.91	0.89	0.90	0.89	0.90
		neutral	0.91	0.89	0.90		0.90	0.88	0.89	
		pro	0.72	0.97	0.83		0.72	0.97	0.83	
MNB	bi-gram	anti	0.92	0.86	0.89	0.83	0.92	0.86	0.89	0.83
		neutral	0.93	0.68	0.79		0.93	0.68	0.79	
		pro	0.51	0.99	0.67		0.51	0.99	0.67	
	tri-gram	anti	0.96	0.57	0.72	0.67	0.96	0.57	0.72	0.67
		neutral	0.95	0.46	0.62		0.96	0.46	0.62	
		pro	0.92	0.97	0.94		0.92	0.96	0.94	
	uni-gram	anti	0.95	0.94	0.94	0.94	0.94	0.94	0.94	0.94
		neutral	0.95	0.91	0.93		0.95	0.91	0.93	
		pro	0.71	0.98	0.82		0.73	0.97	0.83	
LogReg	bi-gram	anti	0.94	0.85	0.89	0.84	0.93	0.86	0.90	0.84
		neutral	0.94	0.69	0.80		0.94	0.69	0.80	
		pro	0.51	0.99	0.67		0.51	0.99	0.67	
	tri-gram	anti	0.95	0.58	0.72	0.67	0.95	0.58	0.72	0.67
		neutral	0.97	0.45	0.62		0.97	0.45	0.62	
		pro	0.93	0.97	0.95		0.92	0.97	0.94	
	uni-gram	anti	0.94	0.94	0.94	0.94	0.94	0.93	0.94	0.94
		neutral	0.95	0.91	0.93		0.95	0.91	0.93	
		pro	0.71	0.97	0.82		0.72	0.97	0.83	
LinearSVC	bi-gram	anti	0.93	0.85	0.89	0.83	0.93	0.87	0.90	0.84
		neutral	0.94	0.69	0.79		0.95	0.69	0.80	
		pro	0.51	0.99	0.67		0.51	0.99	0.67	
	tri-gram	anti	0.95	0.58	0.72	0.67	0.95	0.58	0.72	0.67
		neutral	0.97	0.95	0.61		0.97	0.45	0.62	
		pro	0.93	0.96	0.95		0.93	0.96	0.95	
	uni-gram	anti	0.93	0.97	0.95	0.94	0.93	0.97	0.95	0.95
		neutral	0.96	0.90	0.93		0.97	0.91	0.94	
		pro	0.71	0.98	0.82		0.71	0.98	0.82	
RF	bi-gram	anti	0.93	0.86	0.89	0.84	0.93	0.86	0.90	0.84
		neutral	0.95	0.68	0.79		0.96	0.68	0.79	
		pro	0.51	0.99	0.67		0.51	0.99	0.67	
	tri-gram	anti	0.96	0.58	0.72	0.67	0.96	0.58	0.72	0.67
		neutral	0.98	0.45	0.62		0.98	0.45	0.62	
		pro	0.88	0.93	0.90		0.86	0.93	0.90	
	uni-gram	anti	0.88	0.92	0.90	0.89	0.87	0.92	0.90	0.89
		neutral	0.94	0.84	0.88		0.94	0.82	0.88	
ADA		pro	0.46	0.99	0.63		0.46	0.99	0.63	
	bi-gram	anti	0.90	0.46	0.61	0.60	0.92	0.45	0.60	0.60
		neutral	0.98	0.37	0.53		0.99	0.37	0.54	
		pro	0.39	1.00	0.56	0.48	0.39	1.00	0.56	0.48

	tri-gram	anti	1.00	0.23	0.37		1.00	0.23	0.37	
		neutral	0.97	0.24	0.38		0.97	0.24	0.38	
MLP	uni-gram	pro	0.93	0.96	0.94	0.94	0.93	0.95	0.97	0.94
		anti	0.95	0.95	0.95		0.94	0.94	0.94	
		neutral	0.95	0.92	0.93		0.94	0.92	0.93	
	bi-gram	pro	0.72	0.97	0.83	0.84	0.72	0.97	0.83	0.84
		anti	0.93	0.86	0.89		0.93	0.86	0.90	
		neutral	0.94	0.69	0.79		0.94	0.69	0.80	
	tri-gram	pro	0.51	0.99	0.67	0.67	0.51	0.99	0.67	0.67
		anti	0.96	0.58	0.72		0.96	0.58	0.72	
		neutral	0.97	0.45	0.62		0.96	0.46	0.62	

Table S2. Some notable events throughout the COVID-19 pandemic in Brazil and their dates.

Date	Code	Event	Date	Code	Event
07/10/20	E1	In an online broadcast, the Brazilian President said Brazil joined the Oxford consortium to produce the COVID-19 vaccine.	04/03/21	E24	Brazilian President defends that armed forces apply the COVID-19 vaccine to the population.
07/30/20	E2	Brazilian President speaks about the CoronaVac vaccine.	04/22/21	E25	In an online broadcast, the Brazilian President talked about producing a new drug (nitazoxanide) against COVID-19.
08/06/20	E3	The director of the Butantan Institute[1] says it is possible to have a vaccine for production in October.	04/30/21	E26	Ministry of Health announces commitment to vaccinate the entire Brazilian population by the end of 2021.
08/31/20	E4	The Brazilian President says that the COVID-19 vaccination will not be mandatory.	05/22/21	E27	President says Brazil will have a monthly record for distributing the COVID-19 vaccines in May.
09/10/20	E5	In an online broadcast, the Brazilian President questions the COVID-19 vaccine.	06/02/21	E28	The Brazilian President emphasizes vaccination and criticizes isolation in an official statement.
09/18/20	E6	Government announces its intention to join the COVAX initiative.	06/16/21	E29	In an online broadcast, the Brazilian President

[1] The Butantan Institute, in São Paulo (São Paulo, Brazil), is the largest producer of vaccines in Latin America.

09/24/20	E7	Government announces the release of R$ 2.5 billion for the COVAX initiative.
10/21/20	E8	Brazilian President speaks about Chinese vaccines.
11/09/20	E9	Brazilian President speaks on the availability of vaccines.
11/10/20	E10	The Brazilian President speaks on a social network about the suspension of tests on the COVID-19 vaccine.
12/16/20	E11	Official televised launch of the COVID-19 National Vaccination Campaign
12/18/20	E12	The Brazilian President speaks about the application of vaccines to the Brazilian population.
12/24/20	E13	Online broadcast of the Brazilian President talking about COVID-19 and the vaccination.
12/28/20	E14	The Brazilian President says that laboratories must carry out vaccine registrations to sell to Brazil.
		talks about the contamination of COVID-19 and vaccines.
06/19/21	E30	Brazil surpasses 500 thousand deaths per covid-19.
09/01/21	E31	The Brazilian President criticizes a particular vaccine.
07/07/21	E32	At COVID's Parliamentary Inquiry Commission, a deponent who allegedly lied receives a prison order.
07/18/21	E33	After days of hospitalization, the Brazilian President left the hospital and reinforced the use of drugs against the COVID-19
08/12/21	E34	In an online broadcast, the Brazilian President talks about deaths from COVID-19 after the second shot of the vaccine
08/26/21	E35	Minister of Health praises COVID-19 vaccination, highlighting the national production of the immunizing agents.
08/28/21	E36	Brazil surpasses 60 million people vaccinated, according to the national press consortium's bulletin.
09/15/21	E37	City Hall of Rio de Janeiro makes it mandatory to present a COVID-19 vaccination passport to enter places of collective use.

01/07/21	E15	In an online broadcast, the Brazilian President says he seeks awareness against COVID-19.	09/16/21	E38	The Brazilian President says he will not be vaccinated for a UN event in an online broadcast.
01/17/21	E16	The first COVID-19 vaccine was applied in Brazil.	09/21/21	E39	Brazilian President speaks at the official opening of the 76th UN General Convention and talks about COVID-19 and vaccination in Brazil.
01/22/21	E17	Brazilian President says there is nothing scientifically proven about CoronaVac	09/30/21	E40	The Brazilian President speaks, in an interview, about the use of a vaccination passport in Brazil.
01/25/21	E18	The Brazilian President says the government has approved private companies' purchase of the COVID-19 vaccine.	10/20/21	E41	The proposal for a Report of the COVID-19 Parliamentary Inquiry Commission by the Brazilian Senate is presented on a national broadcast.
02/04/21	E19	Online transmission of the Brazilian President with the President-Director of the National Health Surveillance Agency talking about the COVID-19 vaccination campaign.	10/21/21	E42	Brazilian President speaks about vaccines and COVID kit
02/24/21	E20	On a trip to the state of Acre, the Brazilian President said that whoever gives the final word on Pfizer's vaccine will assume responsibility.	10/24/21	E43	Brazilian President makes an association between the COVID-19 vaccine and AIDS.
03/02/21	E21	Enacted the law authorizing the Federal Government to join the Covax Facility.	10/29/21	E44	Senators present projects making the vaccination certificate mandatory
03/04/21	E22	Brazilian President confirms purchase of vaccine from Pfizer and says that first shots will arrive in April.	10/30/21	E45	At the opening of the G20 meeting, the Brazilian President speaks about the pace of the voluntary COVID-19 vaccination in Brazil.

03/23/21	E23	A presidential statement on the radio announced that Brazil would be self-sufficient in producing COVID-19 vaccines.	

Supplementary Figures and Related Comments

Random Forests (RF) presented the best overall performance, but now, alongside LinearSVC and MLP, appears the LogReg model, with weighted scores very close. Until this point, the aggregated results again corroborate RF as the best model for the multilabel case. Figure S1 presents the learning curves for RF with Count Vectorizer and using uni-grams.

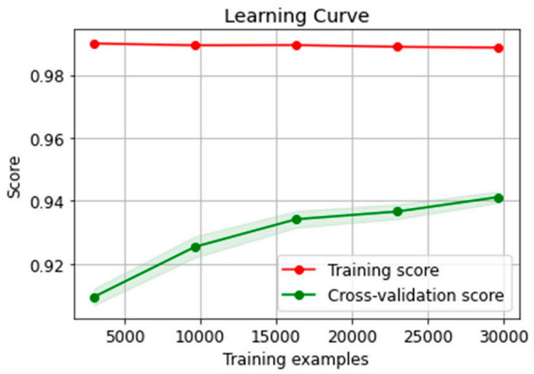

Figure S1. Learning curves for RF with Count Vectorizer and uni-grams.

In the RF training curve, a soft increase in score can be noted by adding more examples, while the increase in cross-validation is more accentuated. Even so, with 30,000 examples, the results did not converge as desired, and these results corroborate the presence of overfitting. The confusion matrix and ROC curves for RF with Count Vectorizer and uni-grams are presented in Figure S2.

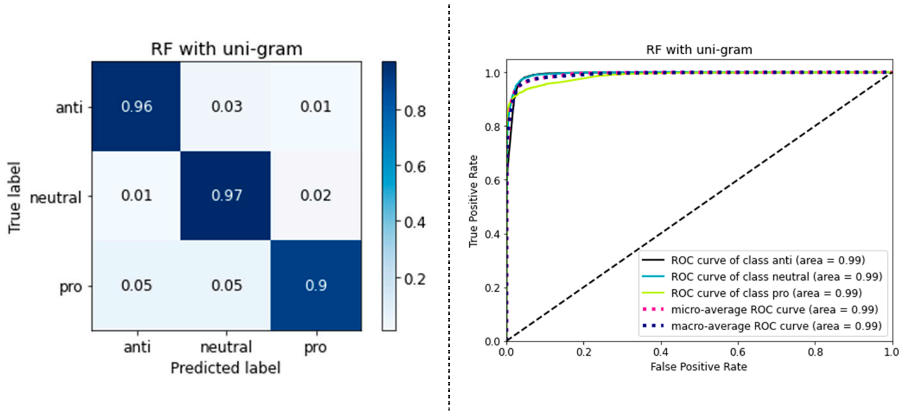

Figure S2. Confusion matrix and ROC Curves for RF with Count Vectorizer and uni-grams.

Once again, RF presented the best overall performance, but now, alongside LinearSVC and MLP, appears the LogReg model, with weighted scores very close. Until this point, the aggregated results again corroborate RF as the best model for the multilabel case. Figure S3 presents the learning curves for RF with Count Vectorizer and using uni-grams.

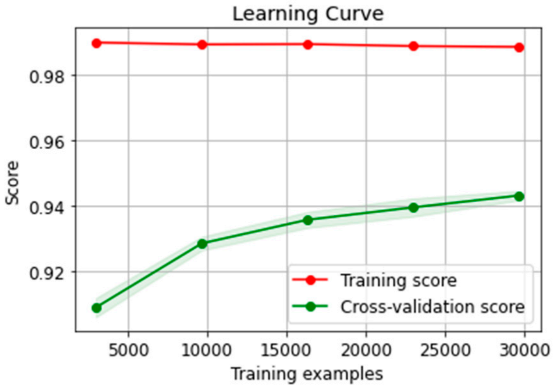

Figure S3. Learning curves for RF with TF-IDF Vectorizer and uni-grams.

In the RF training curve, a soft increase in score can be noted by adding more examples, while the increase in cross-validation is more accentuated. Even so, with 30,000 examples, the results did not converge as desired, and these results corroborate the presence of overfitting. The confusion matrix and ROC curves for RF with TF-IDF Vectorizer and uni-grams are presented in Figure S4.

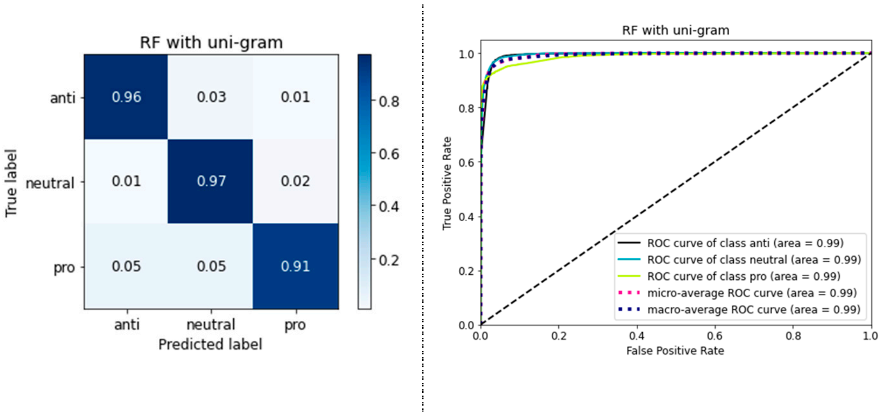

Figure S4. Confusion matrix and ROC Curves for RF with TF-IDF Vectorizer and uni-grams.

True Positive percentages were high for the three classes, with ROC curves demonstrating excellent models, with very high AUCs showing 99% of correct classifications. These excellent performances in classification corroborate even more overfitting in this model.
